# Nationwide Outcome of Gastrectomy with En-Bloc Partial Pancreatectomy for Gastric Cancer

**DOI:** 10.1007/s11605-019-04133-z

**Published:** 2019-02-28

**Authors:** L. R. van der Werf, W. J. Eshuis, W. A. Draaisma, B. van Etten, S. S. Gisbertz, E. van der Harst, M. S. L. Liem, V. E. P. P. Lemmens, B. P. L. Wijnhoven, M. G. Besselink, M. I. van Berge Henegouwen

**Affiliations:** 1 Department of Surgery, Erasmus University Medical Center, Rotterdam, the Netherlands; 2 Department of Surgery, Cancer Center Amsterdam, Amsterdam University Medical Centre, University of Amsterdam, Amsterdam, the Netherlands; 3 Department of Surgery, Meander Medical Centre, Amersfoort, the Netherlands; 4 Department of Surgery, University Medical Centre Groningen, Groningen, the Netherlands; 5 Department of Surgery, Maasstad Hospital, Rotterdam, the Netherlands; 6 Department of Surgery, Medisch Spectrum Twente, Enschede, the Netherlands; 7 Department of Pubic Health, Erasmus University Medical Centre, Rotterdam, the Netherlands; 8 Department of Research, Comprehensive Cancer Organisation the Netherlands, Utrecht, the Netherlands

**Keywords:** Partial pancreatectomy, Gastric cancer, Gastrectomy, R0 resection, Multiviceral resection

## Abstract

**Background:**

Radical gastrectomy is the cornerstone of the treatment of gastric cancer. For tumors invading the pancreas, en-bloc partial pancreatectomy may be needed for a radical resection. The aim of this study was to evaluate the outcome of gastrectomies with partial pancreatectomy for gastric cancer.

**Methods:**

Patients who underwent gastrectomy with or without partial pancreatectomy for gastric or gastro-oesophageal junction cancer between 2011 and 2015 were selected from the Dutch Upper GI Cancer Audit (DUCA). Outcomes were resection margin (pR0) and Clavien–Dindo grade ≥ III postoperative complications and survival. The association between partial pancreatectomy and postoperative complications was analyzed with multivariable logistic regression. Overall survival of patients with partial pancreatectomy was estimated using the Kaplan–Meier method.

**Results:**

Of 1966 patients that underwent gastrectomy, 55 patients (2.8%) underwent en-bloc partial pancreatectomy. A pR0 resection was achieved in 45 of 55 patients (82% versus 85% in the group without additional resection, *P* = 0.82). Clavien–Dindo grade ≥ III complications occurred in 21 of 55 patients (38% versus 17%, *P* < 0.001). Median overall survival [95% confidence interval] was 15 [6.8–23.2] months. For patients with and without perioperative systemic therapy, median survival was 20 [12.3–27.7] and 10 [5.7–14.3] months, and for patients with pR0 and pR1 resection, it was 20 [11.8–28.3] and 5 [2.4–7.6] months, respectively.

**Conclusions:**

Gastrectomy with partial pancreatectomy is not only associated with a pR0 resection rate of 82% but also with increased postoperative morbidity. It should only be performed if a pR0 resection is feasible.

**Electronic supplementary material:**

The online version of this article (10.1007/s11605-019-04133-z) contains supplementary material, which is available to authorized users.

## Introduction

The mainstay of curative treatment in gastric cancer is surgery. For patients with resectable gastric cancer of stage II or higher, neoadjuvant or adjuvant chemotherapy is recommended.^1^ A radical resection with tumor-negative resection margins (pR0 resection) is the most powerful predictor of survival.^2,3^

In patients with advanced gastric cancer, en-bloc partial pancreatectomy may be needed to obtain a pR0 resection. However, the benefits of en-bloc partial pancreatectomy should be critically evaluated given the potential for increased morbidity. Routine splenectomy in patients who underwent a D2 gastrectomy did not lead to increased survival.^4–6^ In the past, a gastrectomy with pancreatosplenectomy was regarded as the standard of care for gastric cancer because it was believed that this would increase lymph node yield and thereby improve oncological outcomes. Since two large trials demonstrated that a D2 lymphadenectomy with pancreatosplenectomy increases postoperative morbidity and mortality without any additional beneficial effects on survival,^7–9^ current guidelines recommend a D2 resection without pancreatosplenectomy.^1^ Nowadays, an en-bloc partial pancreatectomy is only indicated for tumors that invade the pancreas.^1^

The aim of this study was to evaluate patient characteristics and outcomes of en-bloc partial pancreatectomies in patients undergoing gastrectomy for gastric cancer in the Netherlands between 2011 and 2015.

## Methods

### Study Population

For this study, the database of the Dutch Upper Gastrointestinal Cancer Audit (DUCA) was used. Participation in this national audit registry is mandatory for all Dutch hospitals that perform oncological upper gastrointestinal surgery. All patients with gastric or oesophageal cancer who are scheduled to undergo resection are included.^10^ In this audit, patient, disease, and treatment characteristics are prospectively collected. Outcomes are registered until 30 days postoperatively or during hospitalization. The completeness of cases registered in the DUCA approached 100% of patients registered in 2013.^10^

**Fig. 1:**
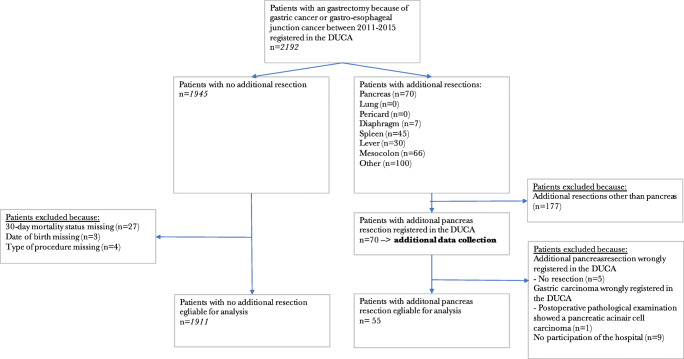
Patients who underwent gastrectomy between 2011 and 2015

Patients who underwent gastrectomy between 2011 and 2015 were selected from the DUCA (Fig. 1). Patients with missing 30-day mortality status (*n* = 27), date of birth (*n* = 3), or type of procedure (*n* = 4) were excluded. When a partial pancreatectomy was registered as an additional surgical procedure, details of patient, treatment, and (long-term) outcome characteristics were provided by participating centers. Patients in whom the partial pancreatectomy was erroneously registered were excluded. For the comparison of patients with and without partial pancreatectomy, patients with other additional resections than pancreatectomy (e.g., splenectomy) were excluded. A separate analysis was executed to compare the occurrence of complications, in patients with partial pancreatectomy compared to patients with other additional (non-pancreas) resections. Another subgroup analysis was executed for patients with a pT4 tumor, the occurrence of complications in patients with partial pancreatectomy was compared to the occurrence of complications in patients without a partial pancreatectomy.

### Outcomes

The prevalence of partial pancreatectomy for gastric cancer was analyzed for all individual hospitals. Characteristics and short-term outcomes of patients with a partial pancreatectomy were evaluated and compared with patients with no additional resection. Also, short-term outcomes were described for both groups: duration of hospital stay, intensive care unit (ICU) stay, resection margins (tumor negative: pR0, microscopically positive: pR1, macroscopically positive: pR2), postoperative complications, postoperative Clavien–Dindo grade ≥ III complications (defined as a complication in combination with a reintervention, readmission to the intensive care unit/medium care unit or death), and 30-day/in-hospital mortality.

Disease-free and overall survival for patients with partial pancreatectomy were evaluated. The following subgroups within the partial pancreatectomy group were compared: pR0 versus pR1 resections and perioperative systemic therapy versus no perioperative systemic therapy.

### Statistical Analysis

Characteristics and short-term outcomes of patients who underwent gastrectomy with and without partial pancreatectomy were compared using Mann–Whitney *U* test and chi-square test, when appropriate. The association between partial pancreatectomy and complications was tested with univariable and multivariable logistic regression analysis. In the multivariable analysis, clinically relevant variables were added to the model, as well as the variables that were associated with complications (*P* value < 0.10 in univariable analyses). The association was tested for sex, age, Charlson comorbidity score,^11^ American Society of Anaestesiologists (ASA) score, tumor location, cT category, and cN category. Overall survival was estimated using the Kaplan–Meier method, and subgroups were compared with log-rank analysis. All analyses were performed using SPSS® version 24 (IBM, Armonk, NY, USA).

## Results

### Patients

Between 2011 and 2015, 2192 patients who underwent a gastrectomy for gastric cancer were registered in the DUCA database. Additional resections were performed in 177 of 2192 patients (8.1%). An additional partial pancreatectomy was performed in 70 of 2192 patients (3.2%) (Fig. 1). The percentage gastrectomies with additional partial pancreatectomy varied between 0 and 10% for the individual hospitals.

Some 55 of 70 patients who underwent additional partial pancreatectomy were included in the analysis because all data could be retrieved from the patient charts. After exclusion of patients with incomplete data, 1911 patients without additional resections served as the control group.

Patient demographics are shown in Table 1. In 12 of 55 patients who underwent a partial pancreatectomy, the tumor was staged preoperatively as cT4. In all 55 patients a preoperative CT scan was performed. In 15/55 (27%) patients, preoperative EUS was performed.

**Table 1:** Patient and disease characteristics of patients undergoing gastrectomy with no additional resection and with additional partial pancreatectomy

	Gastrectomy alone		Gastrectomy plus partial pancreatectomy		*P* value
	*n* = 1911 (97%)		*n* = 55 (2.8%)		
	*n*	%	*n*	%	
Gender					0.53
Male	1207	63%	37	67%	
Female	704	37%	18	33%	
Age (in years, median, IQR)	70	[62–77]	66	[57–73]	
Age (in groups)					0.04
< 65 years	605	32%	22	40%	
65–74 years	645	34%	23	42%	
> 75 years	661	35%	10	18%	
Charlson score					< 0.001
0	835	44%	39	71%	
1	458	24%	7	13%	
2+	618	32%	9	16%	
ASA score					0.71
I-II	1293	68%	39	71%	
III+	600	31%	16	29%	
Unknown	18	0.9%	0	0.0%	
Location of tumor					0.05
Esophageal-gastric junction	69	3.6%	1	1.8%	
Fundus	134	7.0%	8	15%	
Corpus	556	29%	16	29%	
Antrum	771	40%	13	24%	
Pylorus	153	8.0%	9	16%	
Entire stomach	95	5.0%	3	5.5%	
Pouch	59	3.1%	3	5.5%	
Unknown	74	3.9%	2	3.6%	
cT category					< 0.001
cT0–2	571	30%	2	3.6%	
cT3	763	40%	27	49%	
cT4	78	4.1%	12	22%	
cTx	457	24%	14	26%	
Missing	42	2.2%	0	0.0%	
cN category					0.002
cN-0	976	51%	15	27%	
cN+	661	35%	28	51%	
cNx	231	12%	12	22%	
Missing	43	2.3%	0	0.0%	
cM category					0.001
cM-0	1774	93%	49	89%	
cM+	24	1.3%	4	7.3%	
cMx	113	5.9%	2	3.6%	
TNM stage					n.a.
Stage 0	33	1.8%	0	0.0%	
Stage I	392	21%	1	1.8%	
Stage II	637	35%	17	31%	
Stage III	138	8%	8	15%	
Stage IV	24	1.3%	3	5.5%	
Stage unknown	687	36%	26	47%	

*IQR* interquartile range, *ASA* American Society Anaesthesiologists, *n.a.* not available

In the additional pancreatectomy group, total gastrectomy was performed in 31 patients (56%), and 34 patients received perioperative systemic therapy (62%) (Table 2). Additional resections of adjacent organs/structures were performed in 31 of 55 patients, including the spleen (*n* = 25), mesocolon (*n* = 7), liver (*n* = 4), diaphragm (*n* = 1), and other (*n* = 10). Five of 27 patients with a distal pancreatectomy did not undergo a splenectomy. The remaining patients who underwent a splenectomy, *n* = 3, underwent a wedge resection/pancreatic head resection. Upon pathological examination, 34 (62%) tumors were staged as pT4 (Table 2).

**Table 2:** Treatment characteristics of patients undergoing gastrectomy with no additional resection and with additional partial pancreatectomy

	Gastrectomy alone		Gastrectomy plus partial pancreatectomy		*P* value
Treatment	*n* = 1911 (97%)		*n* = 55 (2.8%)		
	*n*	%	*n*	%	
(Neo)adjuvant therapy					0.28
None	779	42%	21	38%	
Neoadjuvant and adjuvant	688	37%	17	31%	
Adjuvant	44	2%	3	6%	
Neoadjuvant	358	19%	14	26%	
Urgency of surgery					0.01
Elective	1833	96%	49	89%	
Urgent/emergency	75	4%	6	11%	
Unknown	3	0%	0	0%	
Curative/palliative					n.a.
Palliative	52	3%	3	6%	
Curative	1835	96%	51	93%	
Prophylactic resection	13	1%	0	0%	
Unknown	11	1%	1	2%	
Type of resection					0.03
Total gastrectomy	803	42%	31	56%	
Partial gastrectomy	1108	58%	24	44%	
Procedure					n.a.
Open	1331	70%	44	80%	
MI abdomen	489	26%	5	9%	
MI abdomen converted	56	2.9%	6	11%	
MI thorax	1	0.1%	0	0.0%	
MI thorax and abdomen	14	0.7%	0	0.0%	
MI thorax and abdomen converted	3	0.2%	0	0.0%	
Unknown	17	1%	0	0%	
Reconstruction					n.a.
No reconstruction	36	2%	1	2%	
Gastric tube	17	1%	1	2%	
Coloninterponate	2	0%	0	0%	
Jejunuminterponate	39	2.0%	0	0.0%	
Esophagojejunostomy	776	41%	30	55%	
Gastro-enterostomy	1007	53%	22	40%	
Other	9	1%	1	2%	
Unknown	25	1%	0	0%	
Additional resections other than pancreatic			31	56%	
Spleen (intentional)			25	45%	
Diaphragm			1	1.8%	
Liver			4	7.2%	
Mesocolon			7	13%	
Other			10	19%	
Pathological T-stage					< 0.001
pT0–2	728	38%	3	6%	
pT3	753	39%	17	31%	
pT4	371	19%	34	62%	
pTx	29	2%	1	2%	
Unknown	30	2%	0	0%	
Annual volume in the hospital or resection					0.20
0–19 resections	1217	64%	37	67%	
20–39 resections	481	25%	16	29%	
40 or more resections	213	11%	2	4%	

*MI* minimally invasive, *n.a.* not available

### Operations

Nine of 55 patients (16%) underwent pancreatoduodenectomy, 27 (49%) distal pancreatectomy, and 19 (35%) a wedge resection (Table 3). In the vast majority (*n* = 52), the indication for partial pancreatectomy was direct tumor ingrowth into the pancreas. Some 30 of 55 resections were performed by a surgeon with experience in pancreatic surgery. In 6 (11%) procedures, the surgical team was changed for the pancreatectomy.

**Table 3:** Details of the partial pancreatectomies: treatment characteristics

Partial pancreatectomies								
	Total		Pancreatoduodenectomy		Distal pancreatectomy		Minimal/wedge resection	
			*n*	%	*n*	%	*n*	%
	55		9	16%	27	49%	19	35%
Indication pancreas resection								
Tumor growth in pancreas	52	95%	9	100%	25	93%	18	95%
Intraoperative injury pancreas	0	0.0%	0	0.0%	0	0.0%	0	0.0%
Lymph node dissection	3	5.5%	0	0.0%	2	7.4%	1	5.3%
Other	0	0.0%	0	0.0%	0	0.0%	0	0.0%
Type of surgeon								
Surgeon with expertise in pancreassurgery^a^	30	55%	7	78%	14	52%	9	47%
Surgeon with expertise in upper GI surgery	25	46%	2	22%	13	48%	10	53%
Change in surgical team								
No	49	89%	8	89%	24	89%	17	90%
Yes, preoperative	2	3.6%	0	0.0%	1	3.7%	1	5.3%
Yes, intraoperative	4	7.3%	1	11%	2	7.4%	1	5.3%
Type of reconstruction								
No	45	82%	3	33%	24	89%	18	95%
Pancreatico-jejunostomy, hepato-jejunostomy, and gastro-jejunostomy	8	15%	6	67%	2	7.4%	0	0.0%
Other	2	3.6%	0	0.0%	1	3.7%	1	5.3%
Drain in pancreatic region (intraoperative)								
Yes	16	29%	4	44%	5	19%	7	37%
No	39	71%	5	56%	22	82%	12	63%
Drain in pancreatic region (postoperative, percut.)								
Yes	45	83%	7	78%	23	85%	15	83%
No	9	17%	2	22%	4	15%	3	17%

^a^In the last year

A pR0 resection was achieved in 45 of 55 patients undergoing gastrectomy with partial pancreatectomy (82%) (Table 4). This was not statistically significant different from the patients who underwent a gastrectomy without additional resection (1617 of 1911, 85%, *P* = 0.82).

**Table 4:** Short-term outcomes of patients with no additional resections versus patients with additional partial pancreatectomies

	Gastrectomy alone		Gastrectomy plus partial pancreatectomy		*P* value
	*n* = 1911 (97%)		*n* = 55 (2.8%)		
	Mean	Median [IQR]	Mean	Median [IQR]	
Hospital stay (days)	14	9 [7–13]	23	14 [10–20]	< 0.001
IC stay (days)	1.8	0 [0–1]	1.8	1[0–2]	> 0.05
	*n*	%	*n*	%	*P* value
Intraoperative complication	73	3.8%	1	1.8%	0.44
Postoperative complication	703	37%	33	60%	< 0.001
Reintervention	279	15%	20	36%	< 0.001
Radiological	83		11		
Endoscopic	38		3		
Reoperation	211		10		
In-hospital and 30-day mortality	101	5.3%	4	7.3%	0.52
Clavien–Dindo grade ≥ III complication	332	17%	21	38%	< 0.001
Resection margins					0.82
R0 Microscopic radical	1617	85%	45	82%	
R1 Microscopic irradical	202	11%	7	13%	
R2 Loco regional residual tumor	25	1.3%	1	1.8%	
Not applicable	21	1.1%	0	0.0%	
Unknown	46	2.4%	2	3.6%	
Multivariable analysis	OR		95% CI		*P* value
Association with Clavien–Dindo grade ≥ III complication^a^					< 0.001
No additional resection	1.00				
Additional partial pancreatectomy	3.13		1.76–5.59		

^a^Adjusted for age, sex, Charlson comorbidity score,^11^ ASA score, location tumor, type of resection (partial/total gastrectomy)

*IC* intensive care, *R0* tumor-negative resection margins, *R1* microscopically tumor-positive resection margins, *R2* macroscopically tumor-positive resection margins, *IQR* interquartile range, *CI* confidence interval, *ASA* American Society Anaesthesiologists

### Complications

In the partial pancreatectomy group, there were relatively more patients with postoperative complications, *n* = 33 (60%) versus *n* = 703 (37%, *P* ≤ 0.001) (Table 4). Also, Clavien–Dindo grade III and higher complications occurred more frequently in the partial pancreatectomy group: in 21 (38%) patients versus 332 (17%) patients (< 0.001). An additional partial pancreatectomy was independently associated with a complication with Clavien–Dindo grade III or higher (OR [95% confidence interval (CI)] 3.28 [1.85–5.82] (Table 4). Postoperative pancreatic fistulas grade B and C according to the International Study Group on Pancreatic Surgery definition were observed in 9 (16%) and 2 (3.6%) patients, respectively (Table 5).^12^ Clavien–Dindo grade III or higher occurred in 42/172 (24%) patients with other additional (non-pancreas) resections; this was not significantly different from the partial pancreatectomy group (38%). For the subgroup of patients with a pT4 tumor, 332/1911 (17%) patients in the gastrectomy only group had a Clavien-Dindo grade III or higher complication versus 4/24 (17%) of patients in the partial pancreatectomy group (*P* = 0.93). Combined in-hospital and 30-day mortality was 7.3% (4 of 53) in patients with partial pancreatectomy versus 5.3% in patients without additional resections (101 of 1911, *P* = 0.52) (Table 4).

**Table 5:** Details of the partial pancreatectomies: treatment characteristics

Pancreatectomies								
	Total		Pancreatoduodenectomy		Distal pancreatectomy		Minimal/wedge resection	
			*n*	%	*n*	%	*n*	%
	55		9	16%	27	49%	19	35%
Postoperative complications								
No	22	40%	2	22%	10	37%	10	53%
Yes	33	60%	7	78%	17	63%	9	47%
POFP^a^								
No POPF, no biochemical leakage	39	71%	6	67%	18	67%	15	79%
No POPF, but biochemical leakage	5	9.1%	1	11%	4	15%	0	0.0%
Yes, grade B	9	16%	2	22%	5	19%	2	11%
Yes, grade C	2	3.6%	0	0.0%	0	0.0%	2	11%
Clavien-Dindo grade ≥ III complication								
No	34	62%	3	33%	19	70%	12	63%
Yes	21	38%	6	67%	8	30%	7	37%
30-day/in-hospital mortality								
No	51	93%	8	89%	26	96%	17	90%
Yes	4	7.3%	1	11%	1	3.7%	2	11%

^a^According to the definition of Bassi&ISGPS, Surgery 2016

*POPF* postoperative pancreatic fistula

### Survival

**Fig. 2:**
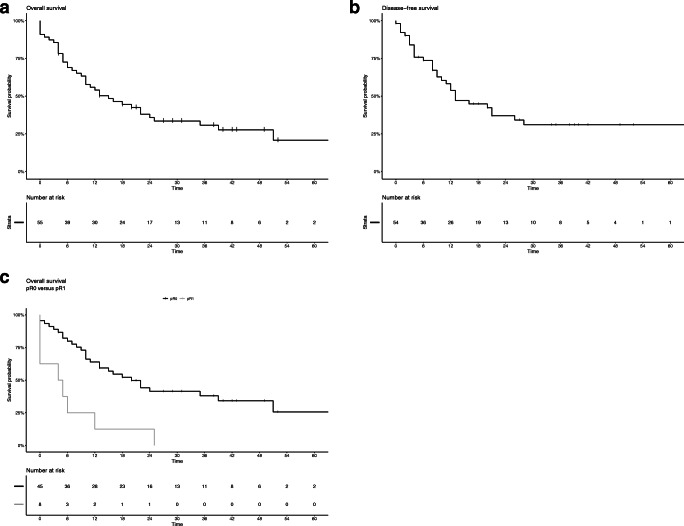
Survival of patients with partial pancreatectomy

Median follow-up of the patients with partial pancreatectomy was 42 [95% CI 36.1–47.9] months. Median overall survival was 15 [6.8–23.2] months (Fig. 2a), and median disease-free survival was 13 [7.6–18] months (Fig. 2b). One-, 2-, and 3-year survival rates were 56%, 38%, and 31%, respectively. In patients in whom an pR0 resection was obtained, median overall survival was 20 [11.8–28.3] months and for patients with an pR1 resection, 5 [2.4–7.6] months (Fig. 2c). For patients treated with perioperative systemic therapy, median overall survival was 20 [12.3–27.7] months versus 10 [5.7–14.3] months for patients without perioperative systemic therapy (Supplementary Fig. 1).

## Discussion

A gastrectomy with en-bloc partial pancreatectomy was rarely performed in the Netherlands between 2011 and 2015. The intraoperative indication for partial pancreatectomy for gastric cancer was usually direct tumor ingrowth in the pancreas. In these patients, additional partial pancreatectomy was associated with an R0 resection rate of 82% but an increased risk for complications.

This study gives a unique overview of the national outcome of patients with gastric cancer for whom an additional partial pancreatectomy was performed during gastrectomy. Most studies on additional resections evaluated different multivisceral resections as one group.^4,13,14^ The national audit database enabled the identification of patients who underwent an additional partial pancreatectomy during a gastrectomy. Because multiple centers participated, we could evaluate the outcomes of a reasonable large cohort of patients treated with gastrectomy with partial pancreatectomy in the Netherlands in the period 2011–2015.

One of the factors associated with improved survival was a radical (pR0) resection. Previous studies also showed a decreased survival in patients in whom an R0 resection could not be achieved.^13–16^ In the present study, the percentage of R0 resections was comparable between the group of patients with partial pancreatectomy and without additional resections (82% versus 85%, *P* = 0.82). In the current literature, the percentages R0 resections after multivisceral resections range from 38 to 100%.^17^ Tran et al. reported an R0 resection rate of 100% in 34 patients after additional partial pancreatectomy.^18^

In this study, only 22% of patients with an additional partial pancreatectomy had a cT4 tumor, and only 62% had a pT4 tumor at pathological examination. Ideally, a partial pancreatectomy should only be performed in actual T4 tumors. In other cohorts with multivisceral resections, low percentages of pT4 tumors have been reported as well (14–80%).^17^ The low percentage of patients with a cT4 tumor shows that there is a discrepancy in the diagnostic assessment of tumor stage with the intraoperative assessment. In order to distinguish a cT3 tumor from a cT4 tumor in the preoperative phase, endoscopic ultrasound (EUS), multidetector row computed tomography (MDCT), and magnetic resonance imaging (MRI) are preferred imaging methods.^19^ Also, when it is not known whether there is ingrowth in the pancreas, it may be recommended to perform an EUS, MDCT, or MRI. The results of the DUCA showed that in only 27% of patients EUS is used for diagnostics. The use of MDCT and MRI were not registered in the DUCA.

The low percentage of patients with a pT4 tumor shows that there is a discrepancy in the intraoperative assessment of tumor stage with the actual tumor stage as seen in pathological examination. Intraoperative frozen section biopsy could be used to assess the resection margin and to decide whether an additonal pancreatectomy is needed. However, dissecting through the tumor plane violates the principle of surgical oncology, i.e., en-bloc resection.

In the present study, patients treated with perioperative systemic therapy had better survival. Selection bias might partly explain this difference. A recent study on the use of perioperative therapy in Dutch patients showed that older patients and patients with a higher ASA score had a lower probability for initiation of perioperative therapy.^20^ In the present cohort, the patients who were not treated with preoperative therapy might have been frail patients who were unfit for undergoing preoperative therapy. These patients are probably more likely to die which could have influenced the survival of this group. Furthermore, exclusion for resection of patients that are progressive during perioperative therapy could have occurred. These data are not available in our surgical database. However, based on our results, it may be wise to take the prognosis of patients without perioperative systemic therapy into account. Patients who are not eligible for perioperative systemic therapy may also not benefit from a partial pancreatectomy during gastrectomy.

Since the MAGIC trial, perioperative chemotherapy for gastric cancer gained importance.^21^ Since partial pancreatectomies are associated with high complication rates, it is possible that patients who undergo a partial pancreatectomy cannot be treated with adjuvant therapy. In the Dutch guideline, perioperative chemotherapy is recommended for patients with stage > 1 gastric cancer and are fit enough to undergo chemotherapy.^1^ This study showed that 38% of patients in the pancreatectomy group were not treated with neoadjuvant therapy neither adjuvant therapy. A recent Dutch study showed that patients with postoperative complications had a threefold increased likelihood of not receiving adjuvant therapy.^22^ It might thus be prudent to focus on a more intense neoadjuvant systemic therapy to patients in whom a partial pancreatectomy is considered. In the future, the results of the CRITICS-II may help in choosing the best neoadjuvant therapy. The CRITICS-II trial aims to optimize preoperative treatment by comparing treatment regimens: (1) chemotherapy, (2) chemotherapy followed by chemoradiotherapy, and (3) chemoradiotherapy.^23^

The performance of additional partial pancreatectomy and splenectomy in order to retrieve more lymph nodes abandoned in the past because of its high postoperative morbidity.^8, 9^

The current study showed high postoperative morbidity in gastrectomy patients with partial pancreatectomies. Complications occurred in 60% of patients, and Clavien–Dindo grade III and higher complications in 38% of patients. Tran et al. reported also a significantly higher percentage of Clavien-Dindo grade ≥ III complications for patients with gastric cancer undergoing a gastrectomy with partial pancreatectomy versus gastrectomy without multivisceral resection (33% versus 17%).^18,24^ These results are comparable to pancreatic cancer patients: a recent study reported the postoperative outcomes of partial pancreatectomies for pancreatic cancer in the Netherlands; they showed that 30% of patients had a Clavien–Dindo grade III or higher complication.^25^

The survival rates in our study were comparable to those reported in a recent study by Mita et al. evaluating additional partial pancreatectomies for gastric cancer. They reported a 1-year survival rate of 62% and a 3-year survival rate of 35% (versus respectively 56% and 31% in the present cohort).^26^ Likewise, the 3-year survival rates of patients with pT4 gastric cancer who underwent multivisceral resections are comparable with the outcomes in our cohort.^27^ Compared to the 2-year survival rate of all potentially curative gastric cancer patients in the Netherlands, the survival of this cohort is poor.^28^ Van Putten et al. reported national 2-year survival rates varying between 38 and 50%, depending on the variation in surgical treatment probability between hospitals.

A limitation of this study was that a pancreatectomy for gastric cancer was not common and not all hospitals in the Netherlands participated in the data collection for patients with partial pancreatectomy. All hospitals have been contacted to participate. The hospitals that did not participate indicated that the reason was of a logistical nature (no time). A second limitation was that survival information was not available for the patients with gastrectomy only. Another limitation was that it was not possible to determine the independent influence of individual parameters on survival because the number of patients undergoing partial pancreatectomy was relatively limited. Because of this limited number of patients, no conclusions could be drawn regarding the different types of pancreatectomies.

 In conclusion, the present study showed that a gastrectomy in combination with a partial pancreatectomy might be considered as a valid curative treatment option for gastric cancer. The reported morbidity and mortality after partial pancreatectomy for gastric cancer are at least comparable to rates after partial pancreatectomy for pancreatic cancer. Therefore, despite the high morbidity, it may be worthwhile to perform a partial pancreatectomy in patients with gastric cancer when the tumor is directly invading into the pancreas. It should probably be reserved for patients with a T4 tumor in whom an R0 resection is feasible. Preoperative and intraoperative selection of patients for additional partial pancreatectomy might be the key to success.

## Electronic supplementary material

Supplementary Fig. 1 (DOCX 89 kb)
