# Supplementary material for: Nationwide Outcome of Gastrectomy with En-Bloc Partial Pancreatectomy for Gastric Cancer
Source: J Gastrointest Surg. 2019 Feb 28;23(12):2327–37. doi: 10.1007/s11605-019-04133-z (PMC6877485; doi:10.1007/s11605-019-04133-z)
Supplement: Supplementary file 1 — (DOCX 89 kb) [file 11605_2019_4133_MOESM1_ESM.docx]

**Supplementary tables/figures:**

*Supplementary figure 1: Overall survival perioperative systemic therapy versus no perioperative systemic therapy in patients with additional partial pancreatectomy*
